# The Impact of Long- and Short-Term Strontium Treatment on Metabolites and Minerals in *Glycine max*

**DOI:** 10.3390/molecules24213825

**Published:** 2019-10-23

**Authors:** Agnieszka Hanaka, Sławomir Dresler, Magdalena Wójciak-Kosior, Maciej Strzemski, Jozef Kováčik, Michał Latalski, Grażyna Zawiślak, Ireneusz Sowa

**Affiliations:** 1 Department of Plant Physiology and Biophysics, Institute of Biological Science, Maria Curie-Skłodowska University, Akademicka 19, 20-033 Lublin, Poland; agnieszka.hanaka@poczta.umcs.lublin.pl; 2 Department of Analytical Chemistry, Medical University of Lublin, Chodźki 4a, 20-093 Lublin, Poland; kosiorma@wp.pl (M.W.-K.); maciejstrzemski@umlub.pl (M.S.); 3 Department of Biology, University of Trnava, Priemyselná 4, 918 43 Trnava, Slovakia; jozkovacik@yahoo.com; 4 Children’s Orthopedics Department, Medical University of Lublin, Gębali 6, 20-093 Lublin, Poland; michallatalski@umlub.pl; 5 Department of Vegetable and Herbal Crop, University of Life Sciences, Akademicka 15, 20-950 Lublin, Poland; grazyna.zawislak@up.lublin.pl

**Keywords:** soybeans, Sr^2+^, phytoestrogens, allantoin, chlorophyll content, Ca^2+^

## Abstract

The impact of long-term exposure to Sr^2+^ (LTE, four doses, 43.5 mg Sr^2+^ per pot, with a total of 174 mg Sr^2+^ per pot during the entire period of cultivation) and short-term exposure to Sr^2+^ (STE, one dose, 870 mg Sr^2+^ per pot four days before harvest) on the content of phytoestrogens and allantoin in soybeans were compared. Sr^2+^ accumulation, the effect on the concentration of macroelements, and basic physiology were also analyzed. LTE reduced the content of malonyldaidzin and malonylgenistin in the roots (58% and 50% compared to the control, respectively). STE increased the amount of all isoflavones in the stem and genistein in the leaves and decreased the content of malonyldaidzin and malonylgenistin in the leaves (55% and 48% compared to the control, respectively) and roots (69% and 62% of the control, respectively) as well as genistein and coumestrol in the roots (both 50% compared to the control). Sr^2+^ presence stimulated the accumulation of allantoin in the roots (three-fold higher than in the control), but only STE had similar effects on the shoots. In contrast to LTE, Sr^2+^ was transported extensively from the roots to the leaves under STE. In comparison to the control, LTE resulted in an increase in the Ca content in the stem by 36%, whereas Ca^2+^ accumulation in the leaves, stems, and roots increased by 60%, 80%, and 36%, respectively, under STE. Additionally, a significant accumulation of K was found only in the roots of the LTE group. The chlorophyll content did not differ between the treatments. Overall, the production of phytoestrogens and Sr accumulation were affected by both the applied dose and the duration of exposure to Sr.

## 1. Introduction

Soybeans (*Glycine max* (L.) Merr.) are especially valuable as a source of biological active compounds such as phytic acid [1], sterols [2], saponin [3], isoflavones [4], and lignans [5]. Recently, there has been an increased interest in health-promoting soybean properties, mainly due to the content of isoflavones such as genistein and daidzein [6]. Isoflavones, which belong to the flavonoid group, are derivatives of 3-phenyl-chromen-4-one. These compounds are mainly found as β-glucosides, as 6′-*O*-acetyl-β-glucosides, as 6′-*O*-malonyl-β-glucosides, and as three aglycones: daidzein (50% of total isoflavones), genistein (40%), and glycine (10%). Soybeans and unprocessed soybean products contain less than 2% of aglycone forms [7,8]. Isoflavonoids possess a wide range of biological activity. They reduce the risk of cardiovascular disease; lower cholesterol levels; have antiartherosclerotic, anti-inflammatory, and antiallergic effects [7,9,10,11]; and prevent some types of cancer by promoting the inhibition of cancer cell growth [12]. In addition, isoflavones may act as antioxidants. Genistein has one of the highest antioxidant activities among dietary flavonoids [13].

Isoflavones are referred to as phytoestrogens [14] due to their similarity to estradiol. They bind and activate estrogen receptors and are generally classified as natural selective estrogen receptor modulators [7]. Antiestrogen activity is useful in the prevention and treatment of diseases associated with endogenous estrogen deficiency, such as postmenopausal osteoporosis [15]. Patients with reduced levels of endogenous estrogens suffer from ailments such as hot flushes, cold sweating, rapid heartbeats, mood swings, sleep disorders, and problems with concentration. An interesting alternative to hormone replacement therapy is supplementation with soybean preparations standardized for the content of isoflavones [16,17,18]. Moreover, a frequent effect of menopause is also osteoporosis. With a reduced production of estrogen, osteolysis prevails over osteogenesis, which in turn results in decreased bone mass and deteriorated bone structure. The therapy proposed currently is mostly based on the use of preparations containing calcium (Ca) or strontium (Sr) [19,20]. Therefore, it seems advisable to search for products that contain both isoflavones and the above-mentioned elements, because the synergism of these components could be particularly important in the prevention and control of diseases associated with the postmenopausal period. Recently, Sr has become attractive as an element used in the biofortification of plants and the production of functional food, which can be applied in order to prevent osteoporosis symptoms [21]. However, it should also be noted that Sr can have negative effects on human health depending on its quantity in a diet, and the radioactive form of Sr can lead to serious bone diseases, including bone cancer [22].

The present paper is a continuation of previous research [18,21,23]. Here, for the first time, we studied the effects of long- (LTE) and short-term exposure (STE) of *Glycine max* to Sr in relation to secondary metabolites (in particular, isoflavones) in leaves, stems, and roots in a soil cultivation system. In addition, the level of Sr^2+^ accumulation was evaluated, and its effect on the concentration of some macroelements in soybeans was determined. Moreover, since the content of chlorophyll is regarded as a plant stress indicator and allantoin is suggested to be a stress mitigation compound, these parameters were also monitored.

## 2. Results and Discussion

### 2.1. Leaf Visual Condition, Biomass, and Relative Chlorophyll Content

**Figure 1:**
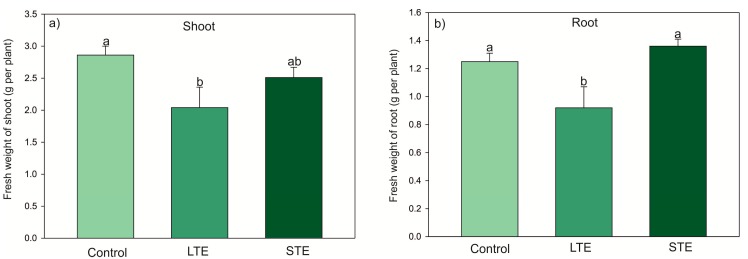
Biomass of (**a**) shoots and (**b**) roots of *Glycine max* in control plants (Control) and under Sr application (long-term (LTE) and short-term (STE) exposure) with standard error (SE) (for each organ, *n* = 5). The data followed by the same letters within the same plants’ organs are not significantly different.

**Figure 2:**
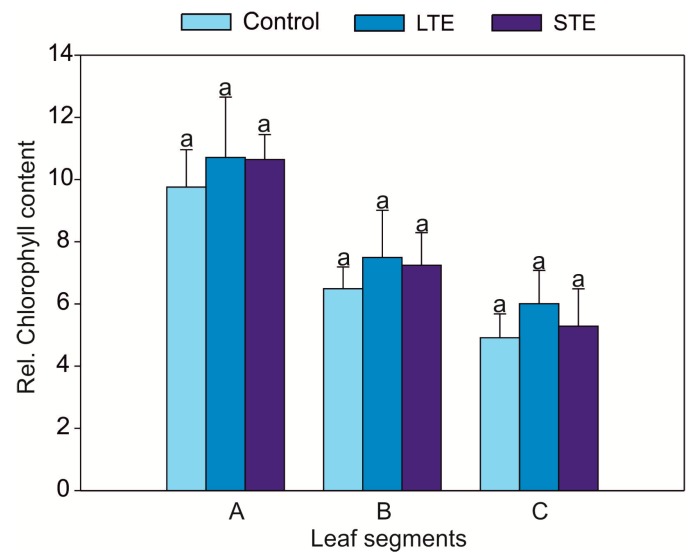
Mean values of the relative chlorophyll content of *Glycine max* in the leaf nodes (I: the closest; II: intermediate; and III: distant from the cotyledon) with standard error (SE). The data followed by the same letters within the same leaf nodes are not significantly different (for each node, *n* = 5). Abbreviations: LTE, long-term exposure to Sr; STE, short-term exposure to Sr.

LTE (four doses of Sr, 43.5 mg = 1 mmol L^−1^ per pot every 11 days, with a total of 174 mg Sr^2+^ per pot during the entire cultivation period (= 4 mmol L^−1^)) did not change the appearance of the soybean plants. However, there was a significantly lower biomass of shoots and roots, reaching 70% and 74% of the weight of the control, respectively (Figure 1). In contrast, STE (single dose of 870 mg Sr = 20 mmol L^−1^ per pot, four days before harvest) caused the necrosis of leaf edges but did not suppress biomass production in shoots and roots (Figure 1). It was found that the relative chlorophyll content decreased in the leaves starting from the first node (the closest to the cotyledon), which exhibited the highest value, and ending with the third node (distant from the cotyledon), which had the smallest value, but the observed changes were not statistically significant. However, the relative chlorophyll content was not a differentiating parameter between the treatments, and thus no effect of Sr^2+^ was found (Figure 2). 

The stable form of the Sr isotope is not considered particularly toxic to plants, and its negative impact on their growth may have been related to a disruption in the uptake of macroelements, mainly Ca [24]. Earlier work has indicated that the presence of a low concentration of Sr in a liquid solution can stimulate soybean growth [21,23] and a high concentration can inhibit soybean growth [21]. In the present study, it was found that LTE reduced the soybean biomass (Figure 1), which to some extent contradicts previous studies [21,23]. However, other reports have indicated that the exposure of higher plants [25,26] and algae [27] to Sr may cause disturbances in biomass production.

### 2.2. Effect of Exposure to Sr on the Content of Phytoestrogens in Soybeans

**Figure 3:**
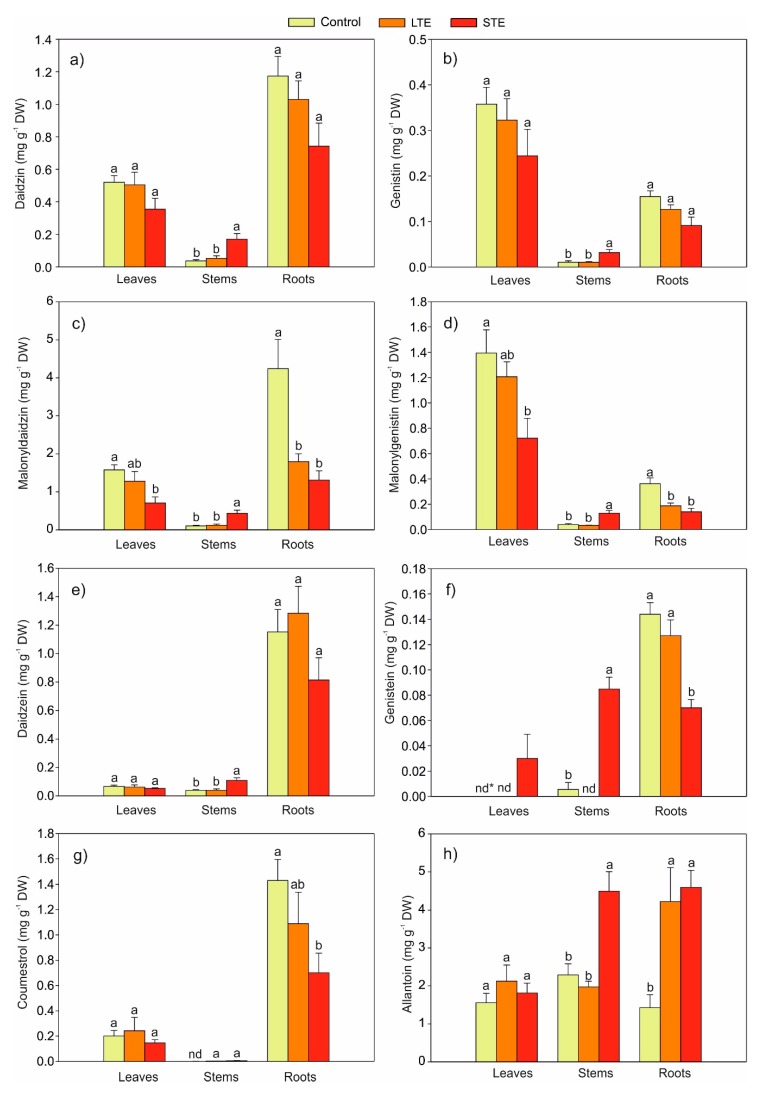
Mean content of phytoestrogens (**a**–**g**) and allantoin (**h**) in *Glycine max* with standard error (SE) (for each organ, *n* = 5). The data followed by the same letters within the same plants’ organs are not significantly different. Abbreviations: LTE, long-term exposure to Sr; STE, short-term exposure to Sr.

Seven phytoestrogens, i.e., six isoflavones (daidzein, daidzin, malonyldaidzin, genistein, genistin, and malonylgenistin) and coumestrol, were identified in soybean extracts. The effect of Sr on the content of aglycons (daidzein and genistein), glucosides (daidzin and genistin), malonylglucosides (malonyldaidzin and malonylgenistin), and coumestrol in the leaves, stems, and roots is shown in Figure 3a–g. It was also shown that malonylglucosides (Figure 3c,d) were the dominant form of phytoestrogens in all Sr treatments and in all tested organs. 

It was found that both LTE and STE significantly affected the content of phytoestrogens in the plant organs. STE significantly induced the accumulation of all isoflavones in the stems and genistein in the leaves compared to the control and LTE. However, STE resulted in a decreased content of malonyldaidzin (Figure 3c) and malonylgenistin (Figure 3d) in leaves (55% and 48% of the control, respectively) and roots (69% and 62% of the control, respectively) as well as genistein (Figure 3f) and coumestrol (Figure 3g) (both ca. 50% of the control) in roots. A similar effect on the content of malonylglucosides in roots was observed in the LTE variant. In comparison to the control, LTE resulted in a reduction of the content of malonyldaidzin and malonylgenistin in the roots by 58% and 50%, respectively. On the other hand, the presence of Sr did not affect the accumulation of aglycones (daidzin, genistin) (Figure 3a,b) and daidzein (Figure 3e) in the leaves and roots and coumestrol (Figure 3g) in the leaves. Considering the sum of phytoestrogens in the individual organs, the negative effect of Sr on the content of the sum of isoflavonoids was as follows: 4.11 (control), 3.62 (LTE), and 2.23 mg g^−1^ (STE) in the leaves and 8.64 (control), 5.62 (LTE), and 3.84 mg g^−1^ (STE) in the roots. However, in the stems, STE increased the sum of isoflavonoids to 1.24 mg g^−1^ (compared to the control (0.23 mg g^−1^) or to LTE (0.25 mg g^−1^)). 

The effects of Sr^2+^ on the content of phytoestrogens in soybeans indicated that the accumulation of these compounds depended on the concentration of Sr^2+^, the age of the plants, and the duration of plant exposure to Sr^2+^ [16,17] or the preincubation of seeds with Sr [17]. Previous studies did not explicitly indicate the effect of Sr on the content of phytoestrogens in soybeans [16,17,23]. Previously, it was noted that the two-week exposure of young soybean plants to Sr^2+^ induced phytoestrogen accumulation. The addition of 2.0 mM Sr^2+^ to the hydroponic medium resulted in an increase in the content of daidzin, coumestrol, genistein, and formononetin by ca. 2.70, 1.92, 3.77, and 2.88 times, respectively [17]. Simultaneously, a further increase in the concentration of Sr^2+^ to the range of 2.5–3.0 mM reduced the accumulation of phytoestrogens to the level observed in the control plants (without Sr^2+^). The contrasting data presented in this study, which indicated that Sr had a negative effect on phytoestrogens in the leaves but not in the stems, showed that this relation is more complicated and is probably evoked by a combination of both the age of the plants and the duration of Sr exposure. This hypothesis was reinforced by a study where the content of isoflavones in soybean was investigated in a 12-week hydroponic experiment using various Sr^2+^ doses (0, 0.5, 2.0, and 4.0 mM Sr^2+^) [23]: it was found that exposure to Sr^2+^, depending on its concentration, reduced the sum of phytoestrogens by 25–70% in individual organs. Moreover, some authors have indicated that the duration of metal stress modifies the accumulation of flavonoids in plant tissues [28]: *Echium vulgare* showed that long-term exposure to heavy metals reduced the content of flavonoids, while short-term exposure induced the accumulation of rutin [28]. The data from the present experiment therefore indicate that changes in the content of metabolites are not just a simple function of the concentration of the stressor applied: other factors such as pretreatment, cultivation conditions, the duration of exposure, and the plant ontogenetic stage also play a role. 

The presented studies revealed that LTE reduced the content of phytoestrogens in the leaves and roots to a lesser extent than STE. However, it is important that STE produced a five-fold increase in the accumulation of isoflavonoids in the stems compared to the controls or to LTE-cultivated plants. The obtained results were probably not only due to the effect of Sr’s impact on the biochemical processes of the synthesis and degradation of isoflavonoids, but were also due to their possible translocation [29].

### 2.3. The Effect of Exposure to Sr on the Content of Allantoin in Soybean

The presence of Sr in soil in both the LTE and STE groups showed a three-fold increase in the content of allantoin in the roots compared to the control (Figure 3h). In contrast to LTE, a stimulatory effect of STE on the content of allantoin was noted in the stem. However, the addition of Sr to the soil did not alter the content of allantoin in the leaves.

The content of allantoin in plants [30] is significantly determined by stress factors [28]. Its increase has been detected in response to solar radiation, drought, salinity, and heavy metals [31,32]. Allantoin is involved in the alleviation of the environmental stress of plants [33]. Its role in increasing tolerance to stress is combined with enzymatic and nonenzymatic antioxidant mechanisms [34]. Nourimand and Todd [34] have suggested that allantoin is an important element activating antioxidant enzymes: superoxide dismutase, catalase, and ascorbic peroxidase. Other studies have confirmed that exogenous allantoin significantly induces the accumulation of antioxidant compounds (ascorbic acid and glutathione) in cucumber under cadmium stress [33]. Environmental stress can significantly increase the formation of allantoin in plants, e.g., the roots of *Echium vulgare* grown in heavy metal-contaminated soil accumulated 10-fold more allantoin than control plants did [28]. The long-term exposure of soybean plants to Sr also induced the accumulation of this ureide [23]. Twelve-week exposure to Sr in hydroponics yielded a ca. 2.5-fold increase in the accumulation of allantoin in roots, with similar but smaller differences observed in soybean leaves and stems [23]. 

### 2.4. The Influence of Sr on Its Accumulation and the Content of Selected Macroelements in Soybeans

**Figure 4:**
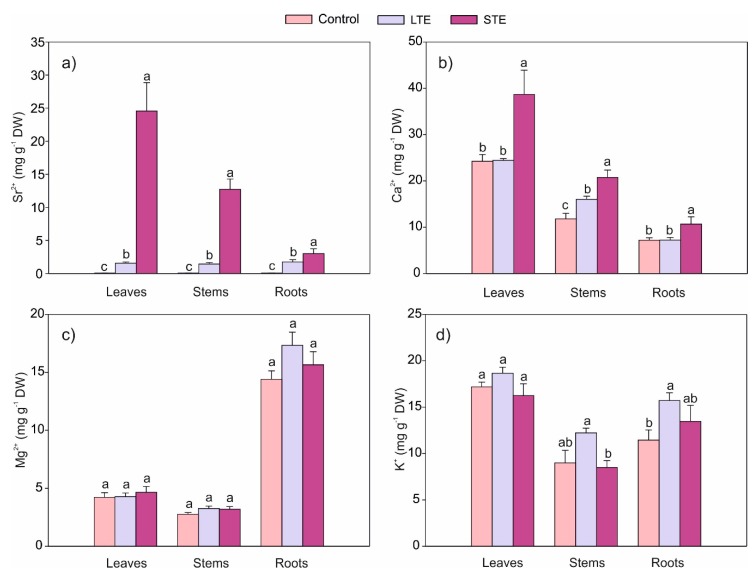
Content of Sr (**a**) and macroelements (**b**–**d**) of *Glycine max* with standard error (SE) (for each organ, *n* = 5). The data followed by the same letters within the same plants’ organs are not significantly different. Abbreviations: LTE, long-term exposure to Sr; STE, short-term exposure to Sr.

The content of Sr^2+^ in individual organs depends on the duration of Sr exposure (Figure 4a). In the case of STE, Sr was very easily transported to aboveground parts, and its accumulation decreased as follows: leaves > stems > roots. This tendency was proven by the translocation factors for leaves to roots and stems to roots (TF*_L_* and TF*_S_*, respectively). In the STE and LTE variants, TF*_L_* was 8.15 and 0.89, respectively, whereas TF*_S_* was 4.42 and 1.01, respectively (Table 1). Moreover, STE caused a significantly higher accumulation of this element (16-, 8.8-, and 1.7-fold in the leaves, stem, and roots, respectively) in comparison to LTE (Figure 4a). 

Soybean is a species with a high potential of Sr^2+^ accumulation [21,35]. The results obtained in the STE variant confirmed the high mobility of Sr (with its easy transfer to aboveground parts), which is consistent with the reports of other authors [21,23]. The intensified accumulation of Sr^2+^ in the shoots of soybean seedlings corresponded with a high translocation factor (from 6.0 to 9.4), which was dependent on the dose of Sr^2+^, and indicated a high potential for biofortification with this element [21]. Dresler et al. [23] have found that translocation factors (TFs) in soybean hydroponic culture are dependent on the dose of Sr and range from 2.6 to 9.3. A high level of TF from roots to leaves for Sr has also been observed in the case of *Sorghum bicolor* grown in soil contaminated with Sr [36] and in plants grown in anthropogenically modified areas [37]. Burger and Lichtscheidl [24] have indicated that Sr is very easily relocated and that the TF is very often above 1. Previous work has confirmed that Sr can be accumulated not only in the leaves, but also in the leaf trichomes [38], stems [39], and bark of coniferous trees [40]. Furthermore, in the LTE variant, we confirmed that Sr was not translocated significantly to the aboveground parts of soybean, as the TF was around 1 or even lower (Table 1).

The present research confirmed that the exposure of the plants to Sr had a significant influence on the Ca content in soybeans (Figure 4b). STE significantly increased Ca accumulation by 60%, 80%, and 36% in the leaves, stems, and roots, respectively, compared to the control. In the case of LTE, a 36% increase in the Ca content was observed only in the stem. These results were surprising because both Sr and Ca move in a similar manner through the same Ca channels, which determines the occurrence of competition and antagonism in the uptake of both elements [41]. Competition during Sr and Ca uptake has been observed in studies in which the presence of Sr in the medium, among other things, inhibited the accumulation of Ca [42,43]. Studies of the influence of Sr on the Ca content in soybean cultivated in hydroponic cultures have indicated some complexity in the uptake and accumulation phenomena of both elements [23]. There, it was found that the long-term exposure of plants to 4 mM Sr decreased the Ca content in the roots and increased its content in the leaves. On the other hand, no relationship was observed in the accumulation of both elements in soybean seeds and stems [23].

The accumulation of Mg was not significantly dependent on Sr exposure, and the content of this element was not significantly different from the control (Figure 4c). At the same time, the translocation of Mg from the roots to the leaves was generally not dependent on Sr supplementation (Table 1). Similarly to the other examined macronutrients, the K content in the soybean leaves was not dependent on the presence of Sr (Figure 4d). In agreement with a previous study where LTE stimulated K accumulation in roots [23], this was the only significant change in the present work.

The observed influence of Sr on macronutrient accumulation in the plants partially contradicted other results [23,42,43], which indicated complexity in the process of macroelement uptake from Sr-rich soil. As demonstrated by Dresler et al. [23], the effect of Sr on the accumulation of elements in plant tissues is a result of the action of many factors, among them the Sr concentration in the medium, the duration of exposure, the plant species, the content of other elements in the medium, and the physical and chemical properties of the medium.

### 2.5. Principal Component Analysis

**Figure 5:**
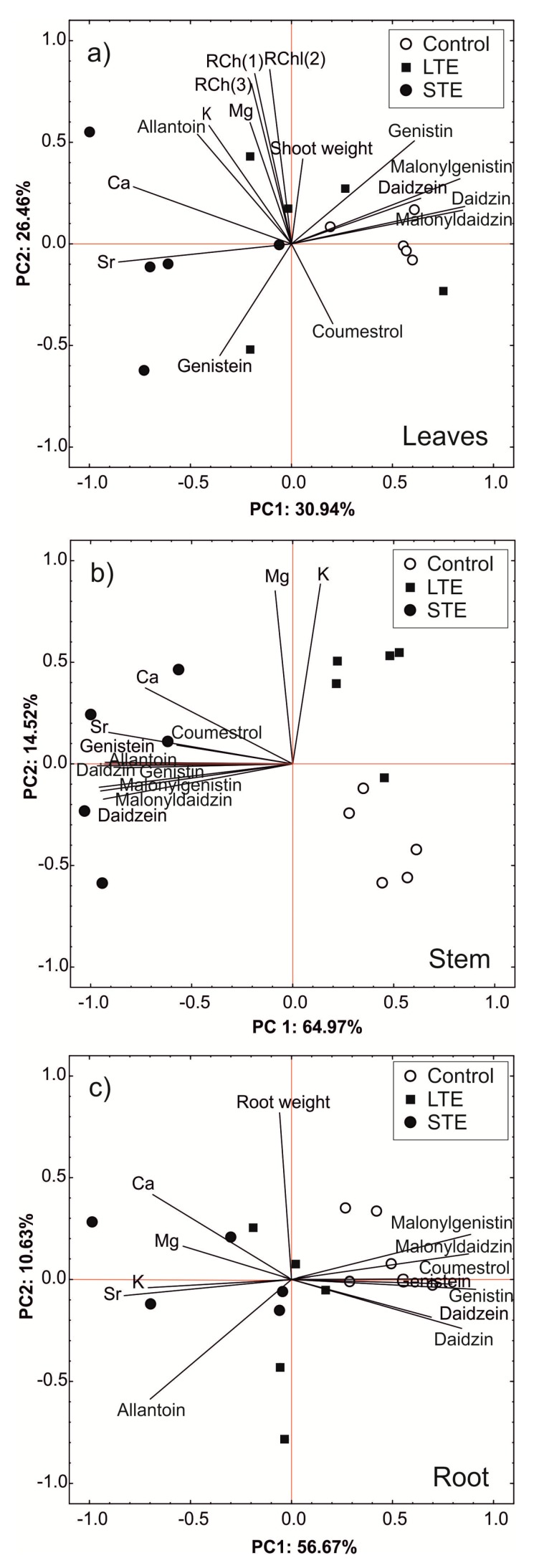
Principal component analysis of *Glycine max* leaves (**a**), stems (**b**), and roots (**c**) (for each organ, *n* = 5). Abbreviations: LTE, long-term exposure to Sr; STE, short-term exposure to Sr; RCh, relative chlorophyll content.

A principal component analysis (PCA) was conducted separately for the leaves, stems, and roots (Figure 5a–c), and a statistical dispersion of the samples is presented. For leaves, the first component and the second component explained 31% and 26% of the total variability, respectively (Figure 5a). The content of phytoestrogens (except genistein and coumestrol), Ca, and Sr were strongly correlated with the first component. Simultaneously, the content of phytoestrogens was negatively correlated with the content of Ca and Sr in the leaves. On the other hand, the content of Mg, K, allantoin, chlorophyll, and genistein as well as the shoot weight were strongly correlated with the second component. A scatterplot of the objects in the space determined by the first two factors indicated a large impact of factor 1 on the grouping of the control objects and their separation from STE leaves. In this case, the control objects were grouped on the right side of the *y* axis, i.e., objects with a high content of malonyldaidzin, daidzin, daidzein, malonylgenistin, and genistein and a low accumulation of Ca and Sr. Objects from the STE treatment, which were characterized by a low content of phytoestrogens and a high content of Sr and Ca, were grouped on the opposite side of the *y* axis.

Figure 5b shows the results of the PCA of the stem samples. The first two factors determined almost 80% of the total variability. The first factor was strongly negatively correlated with the majority of the evaluated variables. In contrast, factor 2 was largely composed of variable Mg and K content. Among the stem samples, there was a strong tendency to group and separate on both sides of the axis of factor 1, leading to a division into two groups: those subjected to STE and both the controls and those subjected to LTE. In contrast to other objects, STE stems accumulated significant amounts of phytoestrogens, Ca, and Sr. On the other hand, factor 2 distinguished between two groups of objects belonging to the control (low content of Mg and K) and a group (with the exception of one object) subjected to LTE (high content of Mg and K).

An analysis of the principal components of the roots displayed that the first two factors determined over 68% of the total variability (factor 1: 57%; factor 2: 11%) (Figure 5c). The first factor was strongly positively correlated with the content of phytoestrogens and was negatively correlated with the content of allantoin and the elements studied. In turn, the second factor was determined by the weight of the roots. In these objects, we observed a clear separation of the control objects (with a high content of phytoestrogens and a low degree of accumulation of allantoin, Sr, and selected macroelements) from objects exposed to Sr (especially in the STE group, where objects with a low content of phytoestrogens but high levels of allantoin and the studied elements were grouped).

## 3. Materials and Methods

### 3.1. Plant Materials

**Figure 6:**
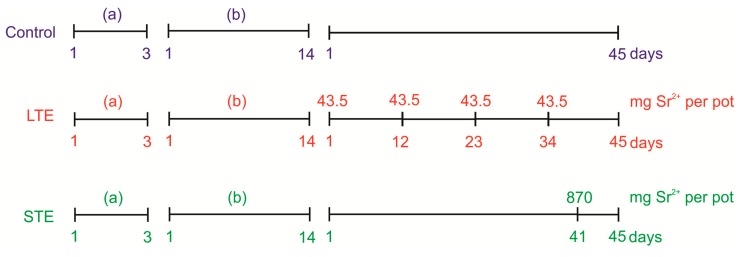
Experimental design: (**a**) seed germination; (**b**) *Glycine max* L. seedling growth in control conditions. LTE: long-term exposure to Sr; STE: short-term exposure to Sr.

Soybean seeds (*Glycine max* L.) were supplied from the Enterprise of Horticulture and Nursery (PNOS, Przedsiębiorstwo Nasiennictwa Ogrodniczego i Szkółkarstwa Sp. z o.o.) in Ożarów Mazowiecki, Poland. The experimental set-up is presented in Figure 6. The seeds were germinated on wet filter paper in a thermostat-controlled chamber for 3 days (at a temperature of 25 °C and with continuous light with a photosynthetic photon flux density of 150 μmol m^−2^ s^−1^). Next, the seedlings were transferred into pots filled with soil (one plant per 0.5-L pot). The chemical composition of the garden soil was as follows: 20.98 N-NH_4_, 137.43 N-NO_3_, 137.68 P-PO_4_, 252.35 K, 2518 Ca, 217.00 Mg, 545. 25 S, and 135.00 Cl (mg dm^−3^ of soil) with a pH of 6.45 and an electrical conductivity of 2.27 mS cm^−1^. After 14 days, the plants were divided into 3 groups (12 plants per treatment): a control (without Sr^2+^), long-term exposure to Sr^2+^ (4 doses at 43.5 mg per pot each time, 1 mmol L^−1^ every 11 days of soil culture (i.e., day 1, 12, 23, and 34)), and short-term exposure to Sr^2+^ (1 dose at 870 mg per pot, 20 mmol L^−1^, exactly at 41 days of soil culture and 4 days before harvest). Strontium was applied as Sr(NO_3_)_2_ (POCH, Gliwice, Poland). After 45 days of soil culture, the plants were harvested and separated into leaves, stems, and roots, which were washed with distilled water and dried with filter paper. The plant parts were weighted and dried at room temperature over 5 days.

The plants were cultivated in a growth chamber equipped with red and blue light-emitting diodes with a photosynthetic photon flux density of 150 μmol m^−2^ s^−1^ under a 16/8-h (day/night) photoperiod at a temperature of 24/18 °C (day/night) with a relative humidity of 70%. During cultivation, the soil moisture in all treatments was kept at the same level.

### 3.2. Leaf Visual Condition, Biomass, and Relative Chlorophyll Content

The leaf visual condition was determined just before the plant harvest. Immediately after the harvest, the plant weight and the relative chlorophyll content were measured. Measurements of the relative chlorophyll content based on dual-wavelength optical absorbance (620 and 940 nm wavelength) were done for leaves of the first, second, and third nodes (distance from the cotyledon) by a Hansatech meter (model CL-01, Hansatech Instruments Ltd., Norfolk, UK).

### 3.3. Sample Preparation

The air-dried samples (divided into leaves, stems, and roots) from 3 plants were mixed, pulverized, and divided into two parts. One part was dried at 80 °C for metal analysis, while the other part was used for an analysis of secondary metabolites. For analyses of phytoestrogens and allantoin, 0.5 g samples were extracted with 5 mL of 80% methanol in an ultrasonic bath for 30 min and centrifuged. Supernatants were extracted with 2.5 mL of 80% methanol (fresh) in an ultrasonic bath for 30 min (twice). For an analysis of metals, 0.5-g samples were digested in a mixture of 3 mL of 65% HNO3 (Suprapur, Merck, Germany) and 7 mL of deionized H_2_O in a microwave digestion apparatus (TOPwave, Analytic Jena AG, Jena, Germany) for 40 min [18].

### 3.4. Analysis of Phytoestrogens

Phytoestrogens were chromatographically determined using high-performance liquid chromatography (HPLC). The procedure was performed on a VWR Hitachi Chromaster 600 (Merck, Darmstadt, Germany) equipped with a gradient pump, a degasser, a thermostat, an autosampler, a diode array detector, and EZChrom Elite software. The separation was carried out in a C18 reverse-phase column LiChrospher 100 (Merck, Darmstadt, Germany) (25 cm × 4.0 mm inner diameter (i.d.), 5 µm particle size) at a temperature of 30 °C. The mobile phase composition was based on the literature data presented in Wójciak-Kosior et al. [17] and consisted of acetonitrile (A) and H_2_O (B) with 0.025% trifluoroacetic acid: the flow rate was 1.5 mL min^−1^. The gradient program, which was based on Szymczak et al. [44], was as follows: A (15%) and B (85%) for 0–5 min; A (20%) and B (80%) for 5–15 min; and A (25%) and B (75%) for 15–40 min. The data were collected in a wavelength range from 200 to 500 nm. The identity of the compounds was established through a comparison of retention times and UV-Vis spectra with corresponding standards purchased from Sigma-Aldrich (St. Louis, MO, USA).

### 3.5. Analysis of Allantoin

The allantoin content was determined using an Agilent 7100 capillary electrophoresis set coupled with a diode array detector (UV-Vis/DAD, 190–600 nm; Agilent Technologies, Santa Clara, CA, USA). The modified method developed by Dresler et al. [45] was applied. The separation was carried out in a fused silica capillary (64.5 cm × 50 µm i.d.) at a temperature of 27 °C. The background electrolyte consisted of a 50-mM borate solution, pH 9.2. A quantitative analysis was performed at λ = 192 nm. Allantoin was identified based on a comparison of retention times and absorption spectrum similarity in a 190–400-nm range with a standard purchased from Sigma-Aldrich (St. Louis, MO, USA).

### 3.6. Analysis of Metals

The concentration of elements was analyzed using a ContrAA 700 high-resolution continuum source atomic absorption spectrometer (Analytic Jena AG, Jena, Germany). A xenon lamp working in an optimized Hot-Spot-Mode and a charge-coupled device (CCD) array detector (185–900 nm) with high quantum efficiency and increased UV sensitivity were employed. TF was calculated based on the ratio of the Sr content in the leaves to the roots (TF*_L_*) and the stems to the roots (TF*_S_*).

### 3.7. Statistical Analysis

The experiment involved three treatments (Control, LTE, and STE), with 12 plants per treatment. The whole experiment was performed two times in the same growth conditions.

The statistical analysis was performed using Statistica ver. 13.3 (TIBCO Software Inc., Palo Alto, CA, USA, 2017). A normal distribution was verified through the Shapiro–Wilk test, and homogeneity was assessed by Levene’s test. The data were analyzed using a one-way analysis of variance (ANOVA), and the significances of differences were examined using a post hoc Tukey test. Data with *p*-values less than 0.05 were considered statistically significant.

A principal component analysis was performed separately for leaves, stems, and roots and was based on the concentration of phytoestrogens, allantoin, metals, plant fresh weight, and relative chlorophyll content.

## 4. Conclusions

The present study showed that even long-term exposure to a five-fold-lower final Sr dose (in comparison to short-term exposure) slightly but significantly depleted plant biomass but had no impact on the chlorophyll content. The quantified isoflavonoids were rather suppressed (or unaltered) by both the STE and LTE treatments in the leaves and roots but not in the stems, indicating that both the applied dose and the duration of the exposure had a significant impact. The calcium accumulation showed a pattern of changes comparable to the Sr uptake pattern, while Sr had almost no impact on K and Mg, which was typical given the relatively low toxicity of Sr (in comparison to other metals). Our data therefore clearly indicate that the application of Sr does not automatically lead to the accumulation of desired metabolites in each organ of soybean.

## Figures and Tables

**Table 1:** Translocation factors (TF) of *Glycine max* of Sr, Ca, Mg, and K for the control, long-term exposure to Sr (LTE), and short-term exposure to Sr (STE) groups (*n* = 5). Data followed by the same letters are not significantly different. Abbreviations: TF*_L_* = TF leaves/roots, TF*_S_* = TF stems/roots.

	Sr	Ca	Mg	K
	TF*_L_*			
Control	-	3.37 ± 0.54 a	0.29 ± 0.06 a	1.50 ± 0.20 a
LTE	0.89 ± 0.14 b	3.38 ± 0.67 a	0.24 ± 0.05 a	1.20 ± 0.18 a
STE	8.15 ± 1.16 a	3.94 ± 0.47 a	0.31 ± 0.06 a	1.25 ± 0.18 a
	TF*_S_*			
Control	-	1.63 ± 0.24 b	0.19 ± 0.04 a	0.79 ± 0.10 a
LTE	1.01 ± 0.27 b	2.21 ± 0.17 a	0.19 ± 0.03 a	0.78 ± 0.15 a
STE	4.42 ± 0.82 a	1.94 ± 0.47 ab	0.20 ± 0.05 a	0.64 ± 0.11 a
